# Association of single nucleotide polymorphisms in Pre-miR-27a, Pre-miR-196a2, Pre-miR-423, miR-608 and Pre-miR-618 with breast cancer susceptibility in a South American population

**DOI:** 10.1186/s12863-016-0415-0

**Published:** 2016-07-15

**Authors:** Sebastián Morales, Felipe Gulppi, Patricio Gonzalez-Hormazabal, Ricardo Fernandez-Ramires, Teresa Bravo, José Miguel Reyes, Fernando Gomez, Enrique Waugh, Lilian Jara

**Affiliations:** Human Genetics Program, Institute of Biomedical Sciences (ICBM), School of Medicine, University of Chile, Av. Independencia 1027, Santiago, Chile; Universidad Andres Bello, Facultad de Ciencias Biológicas, República N°217, Santiago, Chile; Hospital Clínico San Borja Arriaran, Avenida Santa Rosa 1234, Santiago, Chile; Pathology and Oral Medicine, School of Odontology, University of Chile, Sergio Livingstone Pohlhammer 943, Santiago, Chile; National Cancer Society Corporación Nacional del Cáncer CONAC, Santiago, Chile; Clínca Las Condes, Santiago, Chile; Clínica Santa María, Santiago, Chile; Laboratorio de Genética Molecular Humana, Facultad de Medicina, Instituto de Ciencias Biomédicas (ICBM), Programa de Genética, Universidad de Chile, Independencia 1027, Santiago, Chile

**Keywords:** Familial breast cancer, Polymorphisms, MicroRNA, South American population

## Abstract

**Background:**

MicroRNAs (miRNAs) are a novel class of endogenous, non-coding, single-stranded RNAs capable of regulating gene expression by suppressing translation or degrading mRNAs. Single nucleotide polymorphisms (SNP) can alter miRNA expression, resulting in diverse functional consequences. Previous studies have examined the association of miRNA SNPs with breast cancer (BC) susceptibility. The contribution of miRNA gene variants to BC susceptibility in South American women had been unexplored. Our study evaluated the association of the SNPs rs895819 in pre-miR27a, rs11614913 in pre-miR-196a2, rs6505162 in pre-miR-423, rs4919510 in miR-608, and rs2682818 in pre-mir-618 with familial BC and early-onset non-familial BC in non-carriers of *BRCA1/2* mutations from a South American population.

**Results:**

We evaluated the association of five SNPs with BC risk in 440 cases and 807 controls. Our data do not support an association of rs11614913:C > T and rs4919510:C > G with BC risk. The rs6505162:C > A was significantly associated with increased risk of familial BC in persons with a strong family history of BC (OR = 1.7 [95 % CI 1.0–2.0] *p* = 0.05). The rs2682818:C > A genotype C/A is associated with an increased BC risk in non-familial early-onset BC. For the rs895819:A > G polymorphism, the genotype G/G is significantly associated with reduced BC risk in families with a moderate history of BC (OR = 0.3 [95 % CI 0.1–0.8] *p* = 0.01).

**Conclusions:**

The contribution of variant miRNA genes to BC in South American women had been unexplored. Our findings support the following conclusions: a) rs6505162:C > A in pre-miR-423 increases risk of familial BC in families with a strong history of BC; b) the C/A genotype at rs2682818:C > A (pre-miR-618) increases BC risk in non-familial early-onset BC; and c) the G/G genotype at rs895819:A > G (miR-27a) reduces BC risk in families with a moderate history of BC.

## Background

Breast cancer (BC) is the most common cancer among women worldwide. In Chile, BC has the highest mortality rate among cancers (15.8/100,000 women), and its incidence has increased in all age groups analyzed [1]. Genetics factors play an important role in BC development. Currently, there is consensus that mutations in genes BRCA 1 and BRCA 2 are responsible for an average 16 % of the risk for familial BC [2]. It has been proposed that other susceptibility alleles, called moderate or low penetrance, could be responsible for a significant percentage of BC susceptibility. To date, our group has studied the contribution of moderate and low penetrance genes (*PALB2* [3], *BARD1* [4], *ATM* [5], *CHEK 2* [6], *RAD51* [7], *FGFR2* [8], *MAP3K* [8], *TOX 3* [9], 8q24 [9] and 2q35 [9]) to genetic susceptibility for familial BC. Nevertheless, a large part of the genetic component of familial cases remains unidentified [10]. Research on known genes continues in order to further understand BC development, with an emerging interest in epigenetics and gene regulation. One of the most surprising advances in understanding the mechanisms of gene regulation has been the discovery of microRNA (miRNA) [11]. miRNAs are single-stranded RNAs of ~22 nucleotides that can regulate gene expression by either degrading or blocking translation of target miRNA, mainly by binding to their 3’-UTR [12, 13]. MiRNAs are specific to different mRNAs, and approximately 30 % of all human genes are regulated by miRNA [14, 15]. The discovery of miRNAs has been followed by findings highlighting their important and diverse roles in many molecular pathways and biological processes, including development, apoptosis, differentiation, and cell proliferation [16, 17], as well as their implication in various human diseases including cancer. Growing evidence indicates that miRNAs can work as oncogenes or tumor suppressors, depending on which gene(s) they modulate [18]. Atypical expression of various miRNAs has been observed in the development and progression of numerous human cancers [19–21]. Single nucleotide polymorphisms (SNPs) are the most common type of variation in the human genome. SNPs present in the miRNA gene regions can alter expression, lead to maturation to aberrant miRNA, and affect target binding affinity and specificity [22]. Many epidemiological studies have examined the association of miRNA SNPs with cancer susceptibility [19]. In BC, several case–control studies and meta-analyses have evaluated associations between miRNA gene polymorphisms and BC risk in European [23–28], Asian [29, 30], Arab [31], and Jewish [32] populations. With the exception of one study in a Brazilian population [33], the contribution of variant miRNA genes to BC in South American women had been unexplored. In this study, we selected specific SNPs in five miR and evaluated the effects of these SNPs on miR expression and biological function. Recent studies have demonstrated that miR-27a exhibits oncogenic activity by regulating specific transcription factors and the G2-M checkpoint [34–36]. The rs895819:A > G is located at position 40 relative to the first nucleotide of pre-miR-27a [37], and it has been hypothesized that rs895819 could have an effect on the secondary structure of pre-miR-27a, which subsequently affects the processing and/or maturation of miR-27a. Zhang et al. [38] showed that miR-27a expression was significantly lower in BC samples with A/G or G/G genotypes as compared to samples with A/A genotypes, indicating that the A-to-G change decreases expression mature miR-27a. The variant rs11614913, located in the mature miR-196a-3p sequence, could lead to less efficient processing of the miRNA precursor to its mature form and diminish its capacity to regulate target genes such as *HOXB2, HOXB3, HOXC3, HOXB5, GADD45G, INHBB,* and *TP63* [39]. Several studies have shown that miR-423 plays an important role in tumorigenesis [40–42]. In hepatocellular carcinoma, miR-423 promotes cell growth and regulates G(1)/S transition by targeting p21 *Cip1/waf1* [40]. Zhao et al. [43], demonstrated that the SNP rs6505162 in pre-miR-423 affects mature miR expression, and miR-423 plays a potentially oncogenic role in breast tumorigenesis. A few polymorphisms are located in the mature microRNA sequence. Such polymorphisms could directly affect the binding of microRNAs to hundreds of target mRNAs. One of these is rs4919510:C > G, located in mature miR-608. The predicted targets of miR-608 include interleukin-1 alpha (IL1A), growth hormone receptor (GHR), and TP53 [44]. These genes have been reported to be associated with BC [45–47]. A study by Huang et al. [48] showed that the polymorphism rs4919510:C > G in the mature miR-608 sequence contributes to the risk of HER2+ BC. Deregulation of miR-618 has previously been linked to a number of malignancies, including hepatocellular carcinoma [49], male BC [50], and Barrett’s esophageal cancer [51]. Because SNP rs2682818 is part of the miR-618 precursor’s stem-loop sequence, it can affect miR-618 levels. The SNP may alter the secondary stem-loop structure, which in turn influences how pre-miR-618 is processed into its mature form. [52]. Fu et al. [52] suggest that the presence of the variant A allele may negatively impact the production of mature miR-618 by interfering with the post-transcriptional miRNA biogenic process. Considering the proceeding information, in this study we evaluated the association of rs895819 in pre-miR27a, rs11614913 in pre-miR-196a2, rs6505162 in pre-miR-423, rs4919510 in miR-608, and rs2682818 in pre-mir-618 with familial BC and early-onset non-familial BC in non-carriers of *BRCA1/2* mutations from a South American population.

## Methods

### Families

A total of 440 BC cases (one case per family) belonging to 440 high-risk *BRCA1/2-*negative Chilean families were selected from the files of the Servicio de Salud del Area Metropolitana de Santiago, Corporación Nacional del Cáncer (CONAC), and other private services in the Metropolitan Area of Santiago. The majority of the cases are from the Metropolitan Region, and all controls are from the Metropolitan Region. All index cases were tested for *BRCA1* and *BRCA2* mutations as previously described [53]. Pedigrees were constructed on the basis of an index case considered to have the highest probability of being a deleterious mutation carrier. None of the families met the strict criteria for other known syndromes involving BC, such as Li-Fraumeni, ataxia-telangiectasia, or Cowden disease.

Table 1 shows the specific characteristics of the families selected according to the inclusion criteria. All families participating in the study were of self-reported Chilean ancestry dating from several generations, confirmed with extensive interviews with several members of each family from different generations. In the selected families; 16 % (70/440) had cases of bilateral BC; 9 % (40/440) had cases of both BC and ovarian cancer (OC); and 1.1 % (5/440) had male BC. In the BC group, the mean age at diagnosis was 42.1 years, and 75.2 % had age of onset <50 years.

**Table 1:** Inclusion criteria for the families studied

Inclusion criteria	Families: n (%)
Three or more family members with breast and/or ovarian cancer	121 (27.5 %)
Two family members with breast and/or ovarian cancer	148 (33.6 %)
Single affected individual with breast cancer ≤ age 35	87 (19.8 %)
Single affected individual with breast cancer between 36 and 50 years of age	84 (19.1 %)
TOTAL	440 (100 %)

This study was approved by the Institutional Review Board of the School of Medicine of the University of Chile. Informed consent was obtained from all of the participants.

### Control population

The sample of healthy Chilean controls (*n* = 807) was recruited from CONAC files. DNA samples were taken from unrelated individuals with no personal or family history of cancer who consented to anonymous testing. These individuals were interviewed and informed as to the aims of the study. DNA samples were obtained in accordance with all ethical and legal requirements. The control sample was matched by age and socioeconomic strata with respect to the cases.

### Genotyping analysis

Genomic DNA was extracted from peripheral blood lymphocytes of 440 cases belonging to the selected high-risk families and 807 controls. Samples were obtained according to the method described by Chomczynski [54].

Genotyping of the SNPs rs11614913:C > T, rs6505162:C > A, rs895819:A > G, rs2682818:C > A, and rs4919510:C > G was performed using the commercially-available TaqMan Genotyping Assay (Applied Biosystems, Foster City, CA) (assay IDs C__31185852_10, C__11613678_10, C__3056952_20, C__286717_10, and C__2826025_10, respectively). The reaction was performed in a 10-uL final volume containing 5 ng of genomic DNA, 1X TaqMan Genotyping Master Mix, and 1X TaqMan SNP Genotyping Assay. The polymerase chain reaction was carried out in a StepOnePlus Real-Time PCR System (Applied Biosystems, Foster City, CA). The thermal cycles were initiated for 10 min at 95 °C, followed by 40 cycles each of 92 °C for 15 s and 60 °C for 1 min. Each genotyping run contained DNA controls confirmed by sequencing. The alleles were assigned using the StepOne software V2.2 (Applied Biosystems, Foster City, CA). As a quality control, we repeated the genotyping on ~10 % of the samples, and all genotype scoring was performed and checked separately by two reviewers unaware of case–control status.

### Statistical analysis

The Hardy-Weinberg equilibrium assumption was assessed in the control sample using a goodness-of-fit chi-square test (HW Chisq function included in the “HardyWeinberg”.package v.1.4.1). Fisher’s exact test was used to test the association between genotypes and/or alleles for cases and controls. *p* < 0.05 was used as the criterion of significance. Odds ratios (OR) and 95 % confidence intervals (CI) were calculated to estimate the strength of the associations in cases and controls (odds ratio fisher function included in the EpiTools package v.0.5 − 6).

## Results

Selected characteristics of the 440 *BRCA1/2*-negative cases are summarized in Table 1. For the analysis, the whole case sample was subdivided into two groups: cases with two or more family members with BC and/or OC (*n* = 269) (subgroup A) and non-familial early-onset BC (B ≤50 years) (*n* = 171) (subgroup B). The genotype distributions and allele frequencies of the pre-miR-27a rs895819:A > G, pre-miR-196a rs11614913:C > T, pre-miR-423 rs6505162:C > A, miR-608 rs4919510:C > G, and pre-miR-618 rs2682818:C > A polymorphisms in the whole data set and in subgroups A and B with respect to the controls are shown in Table 2. The observed genotype frequencies for four of the five polymorphisms were in Hardy-Weinberg equilibrium in controls (*p* = 0.12 for rs11614913:C > T, *p* = 0.7 for rs6505162:C > A, *p* = 0.3 for rs4919510:C > G, and *p* = 0.8 for rs2682818:C > A, respectively), while for rs895819:A > G the *p*-value was 0.02.

**Table 2:** Genotype and allele frequencies of rs895819, rs11614913, rs6505162, rs4919510 and rs2682818 in *BRCA1/2*-negative breast cancer cases and controls

		All BC cases (*n* = 440)			Families with ≥2 BC and/or OC cases (*n* = 269)			Families with a single case, diagnosed at ≤50 years of age (*n* = 171)		
Genotype or allele	Controls (%) (*n* = 807)	BC cases (%)	*p*-value^a^	OR [95 % CI]	BC cases (%)	*p*-value^a^	OR [95 % CI]	BC cases (%)	*p*-value^a^	OR [95 % CI]
rs895819 (Pre-miR 27a)										
A/A	432 (53 %)	245 (56 %)	-	1.0 (ref)	146 (54 %)	-	1.0 (ref)	99 (58 %)	-	1.0 (ref)
A/G	298 (37 %)	166 (38 %)	0.9	0.9 [0.7–1.2]	105 (39 %)	0.8	1.0 [0.7–1.3]	61 (36 %)	0.5	0.8 [0.6–1.2]
G/G	77 (10 %)	29 (6 %)	0.08	0.6 [0.4–1.0]	18 (7 %)	0.1	0.6 [0.4–1.1]	11 (6 %)	0.1	0.6 [0.3–1.2]
A/G + G/G	375 (47 %)	195 (44 %)	0.4	0.9 [0.7–1.1]	123 (46 %)	0.8	0.9 [0.7–1.2]	72 (42 %)	0.3	0.8 [0.6–1.1]
Allele A	1162 (0.72)	656 (0.75)	-	1.0 (ref)	397 (0.74)	-	1.0 (ref)	259 (0.76)	-	1.0 (ref)
Allele G	452 (0.28)	224 (0.25)	0.1	0.8 [0.7–1.0]	141 (0.26)	0.4	0.9 [0.7–1.1]	83 (0.24)	0.1	0.8 [0.6–1.0]
rs11614913 (Pre-miR 196a2)										
C/C	342 (42 %)	192 (44 %)	-	1.0 (ref)	113 (42 %)	-	1.0 (ref)	79 (46 %)	-	1.0 (ref)
C/T	351 (44 %)	191 (43 %)	0.8	0.9 [0.7–1.2]	127 (47 %)	0.5	1.0 [0.8–1.4]	64 (38 %)	0.2	0.7 [0.5–1.1]
T/T	114 (14 %)	57 (13 %)	0.5	0.8 [0.6–1.2]	29 (11 %)	0.3	0.7 [0.4–1.2]	28 (16 %)	0.8	1.0 [0.6–1.7]
C/T + T/T	465 (58 %)	248 (56 %)	0.6	0.9 [0.7–1.2]	156 (58 %)	0.9	1.0 [0.7–1.3]	92 (54 %)	0.3	0.8 [0.6–1.1]
Allele C	1035 (0.64)	575 (0.65)	-	1.0 (ref)	353 (0.66)	-	1.0 (ref)	234 (0.66)	-	1.0 (ref)
Allele T	579 (0.36)	305 (0.35)	0.5	0.9 [0.8–1.1]	185 (0.34)	0.5	0.9 [0.7–1.1]	120 (0.34)	0.5	0.9 [0.7–1.1]
rs6505162 (Pre-miR 423)										
C/C	284 (35 %)	125 (28 %)	-	1.0 (ref)	74 (28 %)	-	1.0 (ref)	51 (30 %)	-	1.0 (ref)
C/A	385 (48 %)	229 (52 %)	**0.02**	**1.3 [1.0–1.8]**	141 (52 %)	**0.03**	**1.4 [1.0–1.9]**	88 (51 %)	0.2	1.3 [0.9–1.9]
A/A	138 (17 %)	86 (20 %)	**0.05**	**1.4 [1.0–1.9]**	54 (20 %)	**0.05**	**1.5 [1.0–2.3]**	32 (19 %)	0.3	1.3 [0.8–2.1]
C/A + A/A	523 (65 %)	315 (72 %)	**0.01**	**1.4 [1.2–1.8]**	195 (72 %)	**0.02**	**1.4 [1.0–1.9]**	120 (70 %)	0.1	1.3 [0.9–1.8]
Allele C	953 (0.59)	479 (0.54)	-	1.0 (ref)	289 (0.54)	-	1.0 (ref)	190 (0.56)	-	1.0 (ref)
Allele A	661 (0.41)	401 (0.46)	**0.02**	**1.2 [1.0–1.4**]	249 (0.46)	**0.03**	**1.2 [1.0–1.5]**	152 (0.44)	0.2	1.1 [0.9–1.4]
		All BC cases (*n* = 440)			Families with ≥2 BC and/or OC cases (*n* = 269)			Families with a single case, diagnosed at ≤50 years of age (*n* = 171)		
Genotype or allele	Controls (%) (*n* = 807)	BC cases (%)	*p*-value^a^	OR [95 % CI]	BC cases (%)	*p*-value^a^	OR [95 % CI]	BC cases (%)	*p*-value^a^	OR [95 % CI]
rs4919510 (miR 608)										
C/C	431 (53.4 %)	226 (51 %)	-	1.0 (ref)	141 (52 %)	-	1.0 (ref)	85 (50 %)	-	1.0 (ref)
C/G	310 (38.4 %)	174 (40 %)	0.6	1.0 [0.8–1.4]	104 (39 %)	0.8	1.0 [0.7–1.3]	70 (41 %)	0.4	1.1 [0.8–1.6]
G/G	66 (8.2 %)	40 (9 %)	0.5	1.1 [0.7–1.7]	24 (9 %)	0.6	1.1 [0.6–1.8]	16 (9 %)	0.5	1.2 [0.6–2.2]
G/G + C/G	376 (46.6 %)	214 (49 %)	0.5	1.0 [0.8–1.3]	128 (48 %)	0.7	1.0 [0.7–1.3]	86 (50 %)	0.4	1.1 [0.8–1.6]
Allele C	1172 (0.73)	626 (0.71)	-	1.0 (ref)	386 (0.72)	-	1.0 (ref)	240 (0.70)	-	1.0 (ref)
Allele G	442 (0.27)	254 (0.29)	0.4	1.0 [0.9–1.3]	152 (0.28)	0.7	1.0 [0.8–1.3]	102 (0.30)	0.3	1.1 [0.8–1.4]
rs2682818 (Pre-miR 618)										
C/C	699 (86.6 %)	359 (81.6 %)	-	1.0 (ref)	221 (82 %)	-	1.0 (ref)	139 (81 %)	**-**	1.0 (ref)
C/A	102 (12.6 %)	78 (17.7 %)	**0.01**	**1.4 [1.0–2.0]**	45 (17 %)	0.1	1.4 [0.9–2.1]	32 (19 %)	**0.04**	**1.6 [1.0–2.4]**
A/A	6 (0.7 %)	3 (0.7 %)	1.0	0.9 [0.2–3.9]	3 (1 %)	0.4	1.5 [0.3–6.3]	0 (0 %)	0.5	0.3 [0.02–6.8]
C/A + A/A	108 (13.3 %)	81 (18.4 %)	**0.02**	**1.4 [1.0–2.0]**	48 (18 %)	0.08	1.4 [0.8–2.0]	32 (19 %)	0.07	1.4 [0.9–2.3]
Allele C	1500 (0.93)	796 (0.90)	-	1.0 (ref)	487 (0.91)	-	1.0 (ref)	310 (0.91)	-	1.0 (ref)
Allele A	114 (0.07)	84 (0.10)	**0.03**	**1.3 [1.0–1.8]**	51 (0.09)	0.08	1.3 [0.9–1.9]	32 (0.09)	0.1	1.3 [0.9–2.0]

*BC* breast cancer, *OC* ovarian cancer, *OR* odds ratio, *CI* confidence interval

^a^Fisher’s exact test

Bold values are statistically significant (*p* < 0.05)

In the single locus analyses, no significant differences were observed in the genotype and allele distributions for rs11614913:C > T or rs4919510:C > G, either in the whole data set or in subgroups A or B (*p* > 0.05). With respect to rs6505162:C > A, the genotype and allele distribution was significantly different in the whole sample of *BRCA1/2*-negative cases and in subgroup A, with respect to the controls (*p* ≤ 0.05). The minor allele frequency (MAF) (allele A) was higher in subgroup A cases than in controls (0.46 and 0.41, respectively, *p* = 0.03). Furthermore, in subgroup A, allele A carriers (C/A + A/A) had a significantly increased BC risk (OR = 1.4 [95 % CI 1.0 − 1.9] *p* = 0.02) (Table 2). We also analyzed the relationship between rs6505162 and BC risk within cases with a history familial BC according to number of BC cases in the family (Table 3). No association between rs6505162 and BC risk was found in cases belonging to families with two BC and/or OC cases. However, BC risk was significantly higher in cases with three or more family members affected by BC and/or OC. In these families, the allele A frequency was 0.48 in BC cases versus 0.41 in controls (OR = 1.3 [95 % CI 1.0 − 1.7] *p* = 0.04), and homozygous A/A were had a significantly increased BC risk (OR = 1.7 [95 % CI 1.0 − 2.0] *p* = 0.05). No association was found between rs6505162 and non-familial early-onset BC (≤50 years) (Table 2). For rs2682818, located in pre-mir-618, in the whole sample, the MAF (allele A) was higher in cases (0.1) than controls (0.07), and the difference was statistically significant (OR = 1.3 [95 % CI 1.0 − 1.8] *p* = 0.03). This result indicates that allele A is associated with increased BC risk. We also observed increased BC risk for allele A carriers (C/A + A/A) in the whole sample (OR = 1.4 [95 % CI 1.0 − 2.0] *p* = 0.02) (Table 2). When we analyzed the effect of allele A by number of BC cases per family, no association between rs2682818 and BC risk was found. Nevertheless, BC risk increased 1.6-fold in the heterozygous group (OR = 1.6 [95 % CI 1.0 − 2.4] *p* = 0.04) with non-familial early-onset BC (≤50 years) (Table 3).

**Table 3:** Genotype and allele frequencies of rs895819, rs11614913, rs6505162, rs4919510, and rs2682818 by number of BC cases per family, in *BRCA1/2*-negative breast cancer cases and controls

		Families with 2 BC and/or OC cases (*n* = 148)			Families with ≥3 BC and/or OC cases (*n* = 121)		
Genotype or allele	Controls (%) (*n* = 807)	BC cases (%)	*p-*value^a^	OR [95 % CI]	BC cases (%)	*p-*value^a^	OR [95 % CI]
rs895819 (Pre-miR 27a)							
A/A	432 (53 %)	83 (56 %)	-	1.0 (Ref)	63 (52 %)	-	1.0 (Ref)
A/G	298 (37 %)	60 (41 %)	0.8	1.0 [0.7–1.5]	45 (37 %)	0.9	1.0 [0.6–1.5]
G/G	77 (10 %)	5 (3 %)	**0.01**	**0.3 [0.1–0.8]**	13 (11 %)	0.6	1.1 [0.6–2.2]
A/G + G/G	375 (47 %)	65 (44 %)	0.5	0.9 [0.6–1.2]	58 (48 %)	0.7	1.0 [0.7–1.5]
Allele A	1162 (0.72)	226 (0.76)	-	1.0 (Ref)	171 (0.71)	-	1.0 (Ref)
Allele G	452 (0.28)	70 (0.24)	0.1	0.7 [0.5–1.0]	71 (0.29)	0.7	1.0 [0.7–1.4]
rs6505162 (Pre-miR 423)							
C/C	284 (35 %)	41 (28 %)	-	1.0 (Ref)	33 (27 %)	-	1.0 (Ref)
C/A	385 (48 %)	81 (55 %)	0.07	1.4 [0.9–2.2]	60 (50 %)	0.2	1.3 [0.8–2.1]
A/A	138 (17 %)	26 (17 %)	0.3	1.3 [0.8–2.2]	28 (23 %)	**0.05**	**1.7 [1.0–2.0]**
C/A + A/A	523 (65 %)	107 (72 %)	0.08	1.4 [1.0–2.1]	88 (73 %)	0.09	1.4 [0.9–2.2]
Allele C	953 (0.59)	163 (055)	-	1.0 (Ref)	126 (0.52)	-	1.0 (Ref)
Allele A	661 (0.41)	133 (0.45)	0.2	1.2 [0.9–1.5]	116 (0.48)	**0.04**	**1.3 [1.0–1.7]**
rs2682818 (Pre-miR 618)							
C/C	699 (86.6 %)	120 (81 %)	-	1.0 (Ref)	101 (83.5 %)	-	1.0 (Ref)
C/A	102 (12.6 %)	26 (18 %)	0.1	1.3 [0.9–2.3]	19 (15.7 %)	0.3	1.3 [0.7–2.2]
A/A	6 (0.7 %)	2 (1 %)	0.3	1.9 [0.3–9.7]	1 (0.8 %)	1.0	1.1 [0.1–9.6]
C/A + A/A	108 (13.3 %)	28 (19 %)	0.09	1.5 [1.0–2.5]	20 (16.5 %)	0.3	1.2 [0.7–2.1]
Allele C	1500 (0.93)	266 (0.90)	-	1.0 (Ref)	221 (0.91)	-	1.0 (Ref)
Allele A	114 (0.07)	30 (0.10)	0.07	1.4 [0.9–2.2]	21 (0.09)	0.3	1.2 [0.7–2.0]

*BC* breast cancer, *OC* ovarian cancer, *OR* odds ratio, *CI* confidence interval

^a^Fisher’s exact test

Bold values are statistically significant (*p* < 0.05)

The results for rs895819 showed that the homozygous genotype G/G was marginally associated with a protective effect in the whole sample (OR = 0.6 [CI 0.4 − 1.0] *p* = 0.08). Nevertheless, in the families with 2 BC and/or OC cases, we observed decreased BC risk associated with homozygous minor allele genotype (G/G genotype, OR = 0.3 [95 % CI 0.1 − 0.8] *p* = 0.01). This result indicates that the G/G genotype is associated with a protective effect in families with a moderate history of BC.

## Discussion

Mutations in *BRCA1* and *BRCA2* are associated with susceptibility to breast and ovarian cancer. At present, however, those mutations account for only a portion of familial cases, and consequently there is an intensive search for additional targets.

MiRNAs are a class of endogenous, non-coding, single-strand RNAs involved in many molecular pathways and biological processes including apoptosis, differentiation, proliferation, and immune response [55]. SNPs are the most common form of variation present in the human genome. SNPs in miRNA gene regions can affect miRNA function by modulating the transcription of the primary transcript, pri-miRNA and pre-miRNA processing, maturation, or miRNA-mRNA interaction, which could contribute to cancer susceptibility [56]. Recently, many epidemiological studies have examined the association of miRNA SNPs with BC susceptibility, but the results remain inconclusive. Genetic variability is ethnicity-specific, and to date the most miRNA SNP studies have been performed in cases from European, Asian, Arab, and Jewish populations, mainly with sporadic BC. With the exception of one study in a Brazilian population, the role of miRNA variation in BC susceptibility has not been analyzed in a Latin-American population. In the present study, we evaluated the impact of miRNA SNPs on familial and non-familial early-onset BC cases negative for point mutations in *BRCA1/2*, from a Chilean population. To this end, we studied the association of BC risk with rs895819 in pre-miR27a, rs11614913 in pre-miR-196a2, rs6505162 in pre-miR-423, rs4919510 in miR-608, and rs2682818 in pre-mir-618 in a case–control study.

Table 4 shows the results of association studies between SNPs: rs895819 (mir-27a), rs11614913 (miR196a2), rs6505162 (miR-423), rs4919510 (miR-608), rs2682818 (miR-618) and BC risk in others populations.

**Table 4:** Results of association studies of SNPs rs895819, rs11614913, rs6505162, rs4919510 and rs2682818 with BC risk in different populations

		dbSNP			Reference			
n.	MiRNA	(variation)	Phenotype	Author(s)	Country	Ethnicity	N (cases)	N (controls)
1	miR-27a	rs895819	Reduced familial BC	Yang R., et al. (2010) [20]	Germy	Caucasian	1217	1422
				Kontorovich T., et al. (2010) [26]	Israel	Jewish	279	212
				Catucci I., et al. (2012) [18]	Italy	Caucasian	1025	1593
				Zhang M.et al. (2012) [24]	China	Asian	252	248
				Wang B., et al. (2014) [16]	meta-analysis			
2	miR-196a2	rs11614913	Increased BC risk	Hu Z., et al. (2009) [23]	China	Asian	1009	1093
				Hoffman AE., et al. (2009) [51]	USA	Caucasian	439	478
				Catucci I., et al. (2010) [17]	Italy	Caucasian	760	1243
				Catucci I., et al. (2010) [17]	Germany	Caucasian	1134	1517
				Jedlinski DT., et al. (2011) [53]	Australia	Caucasian	187	171
				Alshatwi A., et al. (2012) [25]	Saudi Arabia	Arabian	100	100
				Zhang M.et al. (2012) [24]	China	Asian	252	248
				Linhares JJ., et al. (2012) [27]	Brazil	Brazilian	388	388
				Srivastava K., et al. (2012) [50]	meta-analysis		3449	4140
3	miR-423	rs6505162	Increased BC risk	Kontorovich T., et al. (2010) [26]	Israel	Jewish	279	212
				Smith R., et al. (2012) [57]	Australia	Caucasian	179	174
4	miR-608	rs4919510	Increased HERB2 + BC risk	Huang A-J.et al. (2012) [42]	China	Asian	252	248
5	miR-618	rs2682818	Increased BC risk	Zhang M.et al. (2012) [24]	China	Asian	252	248

Our data do not support an association of rs11614913:C > T and rs4919510:C > G with breast cancer risk. With respect to rs11614913, several case–control studies have been conducted to investigate the association between this SNP with BC susceptibility, but the results have been contradictory. Specifically, case–control studies have shown that rs11614913 SNP is associated with increased BC risk in Han Chinese [29] and Saudi Arabian [57] populations. In contrast, results from studies performed in the United States [58] and China [59] showed that rs11614913 was associated with decreased BC susceptibility. Other studies in Italian, German, and Australian populations reported that the common SNP rs11614913 was not associated with increased BC risk [23, 60]. In Brazilian women with BC, the C/C genotype was associated with decreased BC risk, and the presence of the T allele was significantly associated with increased BC risk [33]. These discrepancies might be explained by different genetic backgrounds. The contemporary Chilean population stems from the admixture of Amerindian peoples with the Spanish settlers in the sixteenth and seventeenth centuries. Later (nineteenth century) migrations of Germans, Italians, Arabs, and Croatians have had only a minor impact on the overall population (not more than 4 % of the total population) and are restricted to the specific locations of the country where they settled [61]. The relationship between ethnicity, Amerindian admixture, genetic markers, and socioeconomic strata has been extensively studied in Chile [62, 63]. Thus, it is probable that in the mixed Chilean population, rs11614913 is not a significant contributor to BC, similar to the results described for Caucasian populations. Another SNP found to have no association with BC risk in our study, rs4919510:C > G, is located in mature miR-608. This is important because few polymorphisms are located in the mature microRNA sequence. Moreover, predicted targets of miR-608 include interleukin-1 alpha (IL-1A), growth hormone receptor (GHR), and TP53 [44], all of which have reported associations with BC. The only case–control study, performed by Huang et al. [48] in Han Chinese women, reported that variant genotypes (C/G + G/G) were specifically associated with increased risk for the HER2-positive subtype in the recessive model, but not for other subtypes. In the Chilean population, we observed no association between this SNP and BC in the whole data set, the familial BC group (subgroup A), or the non-familial early-onset BC group (subgroup B). Nevertheless, our results are not comparable with those obtained in the Han Chinese women as our study did not consider pathologic features of the BC. Further studies in different ethnic groups are needed before concluding whether rs4919510:C > G alters BC susceptibility.

Several studies have evaluated the association between the SNP rs6505162 in pre-miR-423 and cancer risk in diverse populations and in different cancers, with contradictory outcomes. Nevertheless, there have been scarce association studies on this SNP and BC or OC risk. Kontorovich et al. [32] indicated that rs6505162 was associated with a significantly increased risk of ovarian cancer; on the contrary, Smith [64] showed that it conferred a reduced risk of BC. A meta-analysis published by Chen et al. [22] reported no associations between the rs6505162 SNP and BC risk in any genetic model. However, this meta-analysis included only two association studies involving rs6505162 SNP, which is an important limitation to interpreting the results. In our study, we found that the SNP rs6505162:C > A was significantly associated with increased risk of familial BC in the group with a strong family history of BC. In these families, the homozygous genotype A/A was associated with increased BC risk (OR = 1.7 [95 % CI 1.0 − 2.0] *p* = 0.05). Our results are in accordance with the recent results obtained by Zhao et al. [43], who demonstrated that the SNP rs6505162 in pre-miR-423 affects mature miRNA expression and that miR-423 plays a potentially oncogenic role in breast cancer tumorigenesis.

miR-618 deregulation has been related to a number of malignancies, such as hepatocellular carcinoma, [49], male breast cancer [50], and Barrett’s esophageal cancer [51], suggesting a potential rol of this miRNA as a possible cancer biomarker. Because SNP rs2682818 is part of the miR-618 precursor’s stem-loop sequence, it can affect miR-618 levels. The SNP may alter the secondary stem-loop structure, which in turn influences how pre-miR-618 is processed into its mature form [52]. Recently, Fu et al. [52] reported that rs2682818:C > A may play a role in susceptibility to follicular lymphoma (OR = 1.65 [95 % CI 1.05–2.50]).; an in vitro analysis indicated that the variant A allele of rs2682818 lowered mature miR-618 levels. This reduction could trigger a deregulation of miR-618–controlled pathways associated with follicular lymphoma. With respect to BC, the only case–control study published to date reported no association between rs2682818 and BC risk in a Chinese population [30]. Our results showed that the rs2682818 C/A genotype is associated with an increased BC risk both in the whole sample and in the group with non-familial early-onset BC. Our results are the first to contribute to identification of rs6505162 in pre-miR-423 and rs2682818 in pre-miR-618 as polymorphisms associated with increased BC risk in a South American population.

Six studies, including three meta-analyses, have examined the association between the rs895819 polymorphism in miR-27a and BC risk. The studies were conducted in German cases with familial BC, in Italian cases with familial BC, and in Chinese cases with sporadic BC. In the German familial BC cases, the rare (G) allele was shown to have a protective effect limited to cases with age at diagnosis <50 years (OR = 0.83 [95 % CI 0.70 − 0.98] *p* = 0.0314) and bilateral BC (OR = 0.70 [95 % CI 0.52 − 0.95] *p* = 0.0238). The results obtained by Catucci et al. [24] in Italian familial BC failed to support the association of rs895819 with BC risk. In a Chinese population, Zhang et al. [38] showed that in sporadic BC, only younger (<48 years old) allele G (A/G + G/G) carriers showed a significantly reduced BC risk (OR = 0.535 [95 % CI 0.321 − 0.891] *p* = 0.016). With respect to the meta-analyses, the first, which included 4 studies, concluded that subjects carrying the rs895819 G allele showed reduced BC risk [65]. The meta-analysis published by Bai et al. [66] found a significant association between rs895819 allele G and reduced BC risk in Caucasians, but not in Asians. A protective effect of rs895819 allele G was seen in the younger BC cases and in the subgroup of unilateral BC cases. In addition, the meta-analysis published by Chen et al. [22] reported that the miR-27a rs895819 G allele might be a protective factor for BC among Caucasians. Our results in a Chilean mixed population showed that the MAF (allele G) in the controls was low (0.28), similar to the East Asian population [67]. In the whole sample, we observed a marginally protective effect of the genotype G/G, which was likely attributable to SNP frequency and sample sizes. Nevertheless, in the subgroup A, which included families with a moderate BC history, the G/G genotype is significantly associated with reduced BC risk. These results are consistent with the meta-analysis which reported reduced BC risk in Caucasians, as the Chilean population is 60 % Caucasian [68].

## Conclusions

The contribution of miRNA-gene variants to BC susceptibility in South-American women had been unexplored, with the exception of one study in a Brazilian population. Our findings support the following conclusions: a) rs6505162:C > A in pre-miR-423 increases risk of familial BC in families with a strong history of BC; b) the C/A genotype at rs2682818:C > A (pre-miR-618) increases BC risk in non-familial early-onset BC; and c) the G/G genotype at rs895819:A > G (miR-27a) reduces BC risk in families with a moderate history of BC.

## Abbreviations

miRNA, microRNA; SNP, Single Nucleotide Polymorphism; BR, breast cancer; OC, ovarian cancer; OD, odds ratio; CI, confidence interval
